# A two-stage flow-based intrusion detection model for next-generation networks

**DOI:** 10.1371/journal.pone.0180945

**Published:** 2018-01-12

**Authors:** Muhammad Fahad Umer, Muhammad Sher, Yaxin Bi

**Affiliations:** 1 Department of Computer Science and Software Engineering, International Islamic University, Islamabad, Pakistan; 2 School of Computing, Faculty of Computing, Engineering and the Built Environment, Ulster University, Jordanstown Campus, Antrim, United Kingdom; Nankai University, CHINA

## Abstract

The next-generation network provides state-of-the-art access-independent services over converged mobile and fixed networks. Security in the converged network environment is a major challenge. Traditional packet and protocol-based intrusion detection techniques cannot be used in next-generation networks due to slow throughput, low accuracy and their inability to inspect encrypted payload. An alternative solution for protection of next-generation networks is to use network flow records for detection of malicious activity in the network traffic. The network flow records are independent of access networks and user applications. In this paper, we propose a two-stage flow-based intrusion detection system for next-generation networks. The first stage uses an enhanced unsupervised one-class support vector machine which separates malicious flows from normal network traffic. The second stage uses a self-organizing map which automatically groups malicious flows into different alert clusters. We validated the proposed approach on two flow-based datasets and obtained promising results.

## Introduction

**Fig 1:**
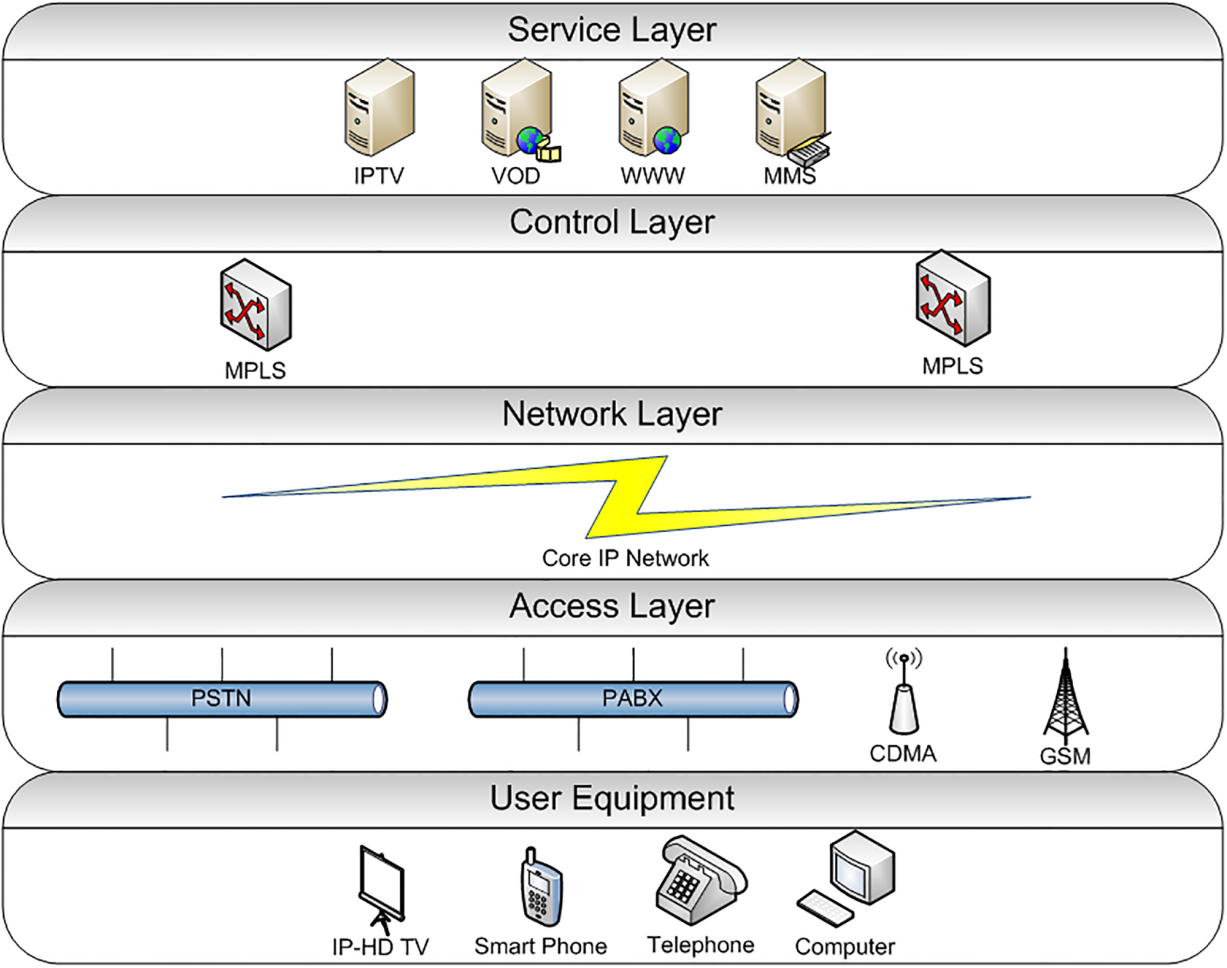
Next-generation network architecture.

A next-generation network (NGN) is an open platform which provides communication, multimedia, and business services through a comprehensive IP-based network architecture. NGN enables the user to use multiple QoS-enabled broadband technologies for service provisioning. These services have been used in multiple business and social applications [1], [2]. The NGN services are provided on converged mobile and fixed networks. The key aspect in a NGN architecture is the separation of service, control, transport and access functions in different layers. These layers are interconnected with each other through well-defined interfaces [3]. Fig 1 shows the architecture of a next-generation network. The user equipment (UE) is connected to the access layer. The access network layer is a combination of legacy networks e.g. PSTN, GSM, and ISDN. The access layer is connected with a core layer. The core layer consists of high-end routers and switches. This layer uses IP network to forward network traffic to a control layer. The control layer comprises of Soft-switches and performs call control and media gateway functions for NGN services. The service provisioning layers include various multimedia and communication NGN services e.g. VoIP, IPTV, VoD and VPN [4].

Next Generation Networks (NGNs) architecture is open for different types of access networks and user services. However, the convergence of heterogeneous network architectures can have serious security implications [5, 6]. Traditional security approaches cannot fully counter the intrusion attacks [7]. To complement traditional security measures, intrusion detection systems (IDS) came in and become an integral part of computer networks. [8] Intrusion detection systems analyze host logs or network activities and raise an alarm if the suspicious behavior is detected.

Despite extensive research in intrusion detection existing [9], a large number of successful cyber attacks on government and corporate intranets have been observed recently. The Global Information Security State Survey [10] gathers that the total number of security incidents detected by respondents climbed to 42.8 million in 2014, an increase of 48% from 2013. The rising trend of attacks shows that existing intrusion detection systems still need improvement, and new approaches are imperative for defense against cyber attacks [11].

The accuracy and efficiency of intrusion detection systems become more important in the context of next-generation networks because the NGN inherits the vulnerabilities of access networks [12]. In this paper, we have proposed a flow-based intrusion detection model for next-generation networks. Our approach uses a two-stage process for detection of malicious activity in network traffic. The first stage of the detection process uses one-class support vector machine (SVM) and determines if a network flow is malicious or normal. Although one-class SVM is supervised learning technique, we employ an enhanced version of one-class SVM which supports unsupervised learning. The second stage process employs a self-organizing map (SOM) to automatically cluster malicious flows in different attack classes. We validated the proposed approach on three flow-based datasets and obtained promising results.

The remainder of this paper is organized as follows. Section 2 gives an overview of flow-based intrusion detection. Section 3 describes the existing work in flow-based intrusion detection systems. Section 4 gives a detail description of our proposed approach. The datasets used for evaluation of the proposed approach are discussed in section 5. We discuss experimental results in section 6. Finally, the conclusion is presented in Section 7.

## Flow-based intrusion detection

Traditional intrusion detection systems use deep packet or state-full protocol inspection to detect malicious activity in the network traffic. Deep packet inspection (DPI) techniques scan the packet beyond the protocol header and inspect its content. The DPI techniques provide complete visibility of network traffic and filter the packet content for malware, virus or any other attack traces. [13] However, deep-packet inspection becomes impractical for high-speed backbone links [14]. Also, the deep-packet inspection is not possible when packet content is encrypted. In state-full protocol inspection, the complete semantics of the protocol are checked against the specification and any out of the range value is considered an anomaly. State-full protocol inspection techniques are protocol specific and cannot be generalized for unknown protocols. Also, both packet and state-full protocol inspection techniques are computationally costly and become a performance bottleneck [9, 15].

Packet and protocol-based intrusion detection systems cannot be used in next-generations networks due to their limitations [16, 17]. An alternative solution for protection of next-generation networks is flow-based intrusion detection [15, 18]. The flow-based intrusion detection systems use network flow records as input and try to find out if network traffic is normal or malicious [19]. The flow records contain aggregated information of related network packets. A network flow is defined as “a set of packets or frames passing an observation Point in the network during a certain time interval. All packets belonging to a particular flow have a set of common properties” [20]. The network flows have a number of applications including network traffic accounting, billing, monitoring and security.

The network flows are collected from the network using a flow export and collection protocol. The process of flow export and collection is controlled by a flow export protocol. The most common flow collection and export protocol is Cisco’s Netflow. Netflow was adapted by IETF and has been formalized in the form of IP Flow Information Exchange (IPFIX) [20]. The deployment of IPFIX/Netflow consists of following processes:-

- *Packet capturing at observation point*. Observation points collect the packets being passed through a specific interface. The observation points can be standalone devices or a part of flow-enabled routers. The observation process forwards the packet to a flow metering and export process.
- *Flow metering and export process*. The metering process time-stamps the packets and aggregates them into network flows. The flows can be sampled or filtered according to the requirements. These flows are forwarded to an export process which exports the flows in IPFIX record. These IPFIX records are forwarded to a collector process.
- *Collecting process*. The collecting process receives IPFIX records from exporting process. There can be multiple collecting processes receiving IPFIX records from different exporting processes. Accordingly there can be multiple exporting processes sending IPFIX records to multiple collecting processes. The collecting process store and pre-process the flow data for the flow analysis and monitoring application.

Flow-based intrusion detection systems are also based on the generic intrusion detection model presented in [21]. The incoming flow records are collected from the network using observation points (event-boxes). These flows can be optionally stored in a flow database (database-boxes). Then these flows are forwarded to an analysis box for evaluation. The analysis box uses anomaly detection techniques for attack detection. If any attack is detected, a response is initiated through the response-boxes.

## Related work

Flow-based intrusion detection is an active area of research. A number of flow-based techniques using statistical and machine learning methods for detection of malicious flows have been proposed. In [22], the authors proposed a flow-based intrusion detection system using SVM based one-class classification. The one-class SVM (OC-SVM) uses malicious flows as target class. Learning on malicious flows is fast and efficient. After learning, the OC-SVM detects the malicious flows while normal flows are discarded. A flow-based dataset developed by [23] is used for evaluation. The OC-SVM gives very good results with 98% accuracy and 0% false alarm rate. These malicious flows can be further analyzed for identification of attack type.

A network anomaly detection system using multiple unsupervised clustering techniques is presented in [24]. The technique uses a change detection algorithm to detect the malicious flows. The malicious flows are clustered in partitions using sub-space and density-based clustering. The clusters are also ranked in order of abnormality and all clusters above the detection threshold are considered anomalies. The technique is evaluated on MAWI and KDD99 datasets and results show that proposed technique obtained good results using unsupervised learning algorithms.

A flow-based intrusion detection technique using block based neural network (BBNN) is proposed in [25]. The BBNN is constructed using Field Programmable Gate Arrays (FPGAs) for efficient and real-time processing of high volume of data. The input to a neuron block in BBNN is a vector of values while output is calculated using sum of the weighting vector value and a bias. The technique is evaluated on NetFlow records generated from the DARPA dataset. The results show that detection rate of BBNN is same as off SVM, but the running time is quite good because of hardware-based detection engine. However, the results are obtained from a packet-based dataset which was manually labeled.

A flow-based anomaly detection system using Principle component analysis (PCA) is proposed in [26]. The sketch data structure is used to store the hash value of network traces. The hashed network traces are converted into entropy time-series and forwarded to a PCA classifier. The technique is evaluated on MAWI dataset [27]. The proposed technique show improvement in results when compared to other PCA based anomaly detectors over the same dataset.

A multi-layer perceptron (MLP) with heuristic optimization algorithm is suggested in [28]. The MLP interconnection weights are optimized using two heuristic techniques: Cuckoo and Particle Swan Optimization with Gravitational Search Algorithm (PSOGSA). The method performs classification of malicious and benign network flows. The technique is evaluated on the dataset used in [22] and flow records generated from DARPA. The results show that MLP with PSOGSA optimization gives the accuracy of 99.55% with 0.21% false alarm rate.

In [29], the authors have proposed a two-stage neural network for intrusion detection using flow records. Two neural network structures, multilayer and radial basis function networks, have been used to compare performance. The first stage detects significant changes in the traffic that could be an attack. If an attack is detected, the flow data is forwarded to a second stage classifier which determines the type of attack. The technique is evaluated on Netflow v5 records generated from DARPA dataset. The first stage neural network gives 94.2% detection rate and 3.4% false positive rate. For second stage, best detection rate of 99.42% is also obtained with a false positive rate of 2.6%.

An improved nature-inspired technique for optimum-path forest clustering (OPFC) is proposed in [30]. The OPFC is a k-NN graph in which nodes are weighted using a probability density function. The authors used different optimization techniques including Bat algorithm, Gravitational search, Harmony search and Particle swarm optimization to determine the best value of k. The approach has been evaluated on a flow-based dataset and results show that optimum-path forest clustering outperforms k-means and SOM in flow-based detection.

A ward clustering approach to detect the dictionary attacks over SSH is presented in [31]. SSH is a very common way to access the remote servers over the Internet and remains a favorite attack target. The authors used two innovations of employing of checking the existence of connection protocols, measure men of auth-packet and the next and identification of transit point of each sub-protocol. The best results include a 99.90% detection rate for unsuccessful SSH attack attempts and 92.80% detection of successful SSH attempts.

Although there is extensive work in flow-based intrusion detection, Our approach significantly differs from the existing work. We have used a multi-stage approach that swiftly discards normal flows in the first stage. The second stage only process malicious flows and no resources are wasted on unnecessary inspection of normal flows. The first stage uses one-class classification with the malicious flow category as positive class. All normal flows are considered outliers. Learning on malicious flows is fast and efficient because the malicious traffic is only in a fraction of normal traffic. The second stage categorizes the flows in the different alert cluster based on the flow characteristics. This provides a deep insight into the malicious traffic and under attack services. Both classification stages use unsupervised learning, therefore, no labeled training set is required. We have evaluated the proposed framework on a realistic flow-based dataset, therefore, experimental results are very close to real-world scenarios.

## Proposed approach

**Fig 2:**
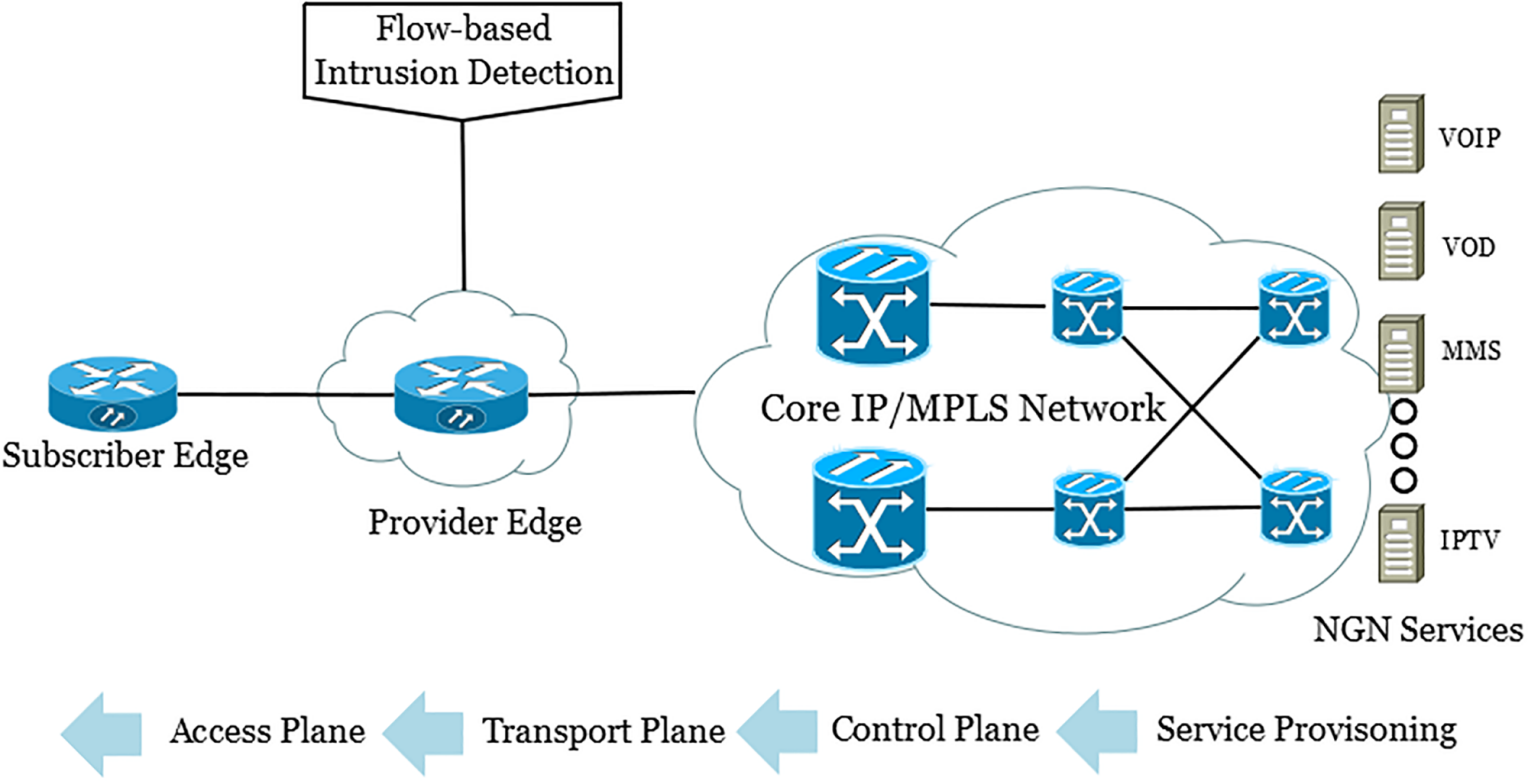
Flow monitoring in next-generation network architecture.

NGN encapsulates a variety of network architectures, services, and protocols in a layered architecture. The IPFIX/Netflow flow records provide a unified way to access traffic flow information from the next-generation network. These flow records are collected from the network using specialized flow-enabled network devices. The flow data is accessed by a flow analysis application for congestion detection, billing and network security. Fig 2 shows the implementation of flow-based intrusion detection system in the NGN framework. The flow information is collected at the provider edge and forwarded to the intrusion detection system. Provider edge is a router installed at the boundary of the network. The intrusion detection system analyzes the flow records passing through the provider edge and raise an alarm if malicious flows are detected.

**Fig 3:**
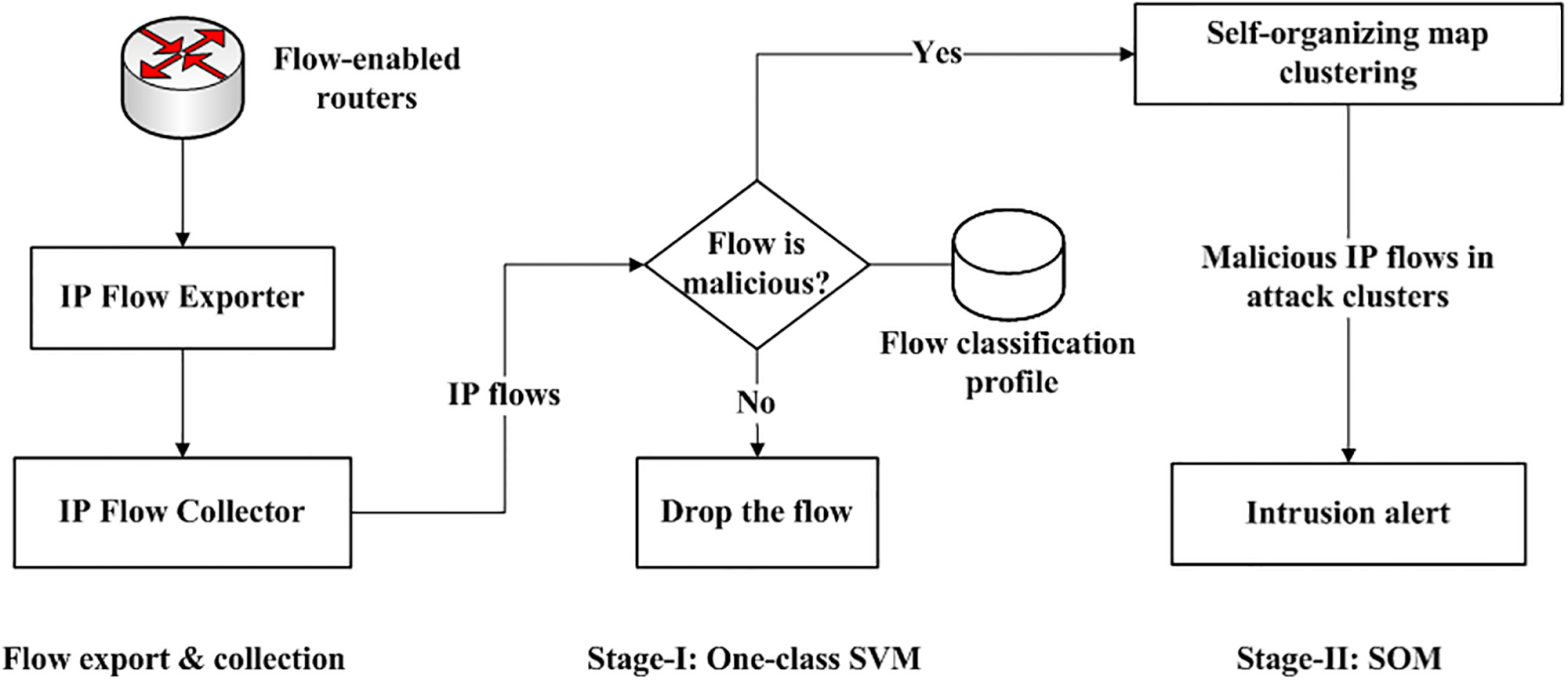
Architecture of two-stage flow-based intrusion detection system.

We propose a two-stage intrusion detection model to detect malicious traffic in next-generation networks using network flows. Fig 3 shows the architecture of our approach. The model analyzes the flow data to detect malicious network traffic. The intrusion detection model consists of two stages. The first stage detection process employs a one-class support vector machine(SVM). The one-class classifier only identifies malicious flows while all other flows are discarded. The malicious flows are forwarded to the second stage which uses self-organizing map to group similar malicious flows into different attack clusters. Every attack cluster represents a specific type of network attack.

### The first stage detection

The first stage detection separates malicious and normal flows network traffic. Since we have only one class of interest i.e. malicious, the problem is solved by using a one-class classifier [32]. The one-class classifier recognizes objects of only one class. All input objects are either belong to a target (positive) class or considered outliers [33]. One-class classification is used when training dataset for only one class (target class) is available. The training datasets for other classes are not available or difficult to obtain.

The one-class classification has already been in use for intrusion detection [34]. Available once class classification includes density estimation, reconstruction methods and support vector machines (SVM). We use SVM-based one-class classification techniques because SVM techniques give accurate results for intrusion detection [35, 36]. One-class SVM constructs a boundary around the target class objects in the form of a hyperplane. The hyperplane is constructed in the feature space such that distance from the origin is maximum [37].

Mathematically, we assume that *x*_*i*_ is a training example from a dataset *X* = {*x*_1_, …, *x*_*m*_} in the input space. Let *ϕ* is a mapping function which maps the input feature space *X* to a high dimensional feature space *H*. The dot product in *H* can be computed using following simple kernel function:-

$$\begin{array}{c} K\left(x,y\right)={\left(\phi \left(x\right).\phi \left(y\right)\right)}_{H} \end{array}$$ (1)

To separate the input examples from the origin with maximum margin using a hyperplane, following quadratic condition is applied

$$\begin{array}{c} min_{w,\xi ,\rho }\frac{1}{2}{\left\|w\right\|}^{2}+\frac{1}{m\nu }\sum_{i=1}^{m}\xi_{i}-\rho \end{array}$$ (2)

Subject to

$$\begin{array}{c} \left(w.\phi \left(x_{i}\right)\right)\geq \rho -\xi_{i},\xi_{i}\geq 0 \end{array}$$ (3)

The *ξ*_*i*_ is a slack variable used to penalize the outliers. The *ρ* is the offset and *w* is weight vector. The *ν* ∈ (0,1) is a user-defined error control parameter and sets an upper bound on the fraction of outliers and a lower bound on the number of support vectors. A function *f*(*x*) is defined which takes the value +1, if *x* falls within the hyperplane and −1 otherwise. Solving the above the minimization problem, the decision function for classification is defined:

$$\begin{array}{c} f(x)=sgn((w.\phi (x))-\rho ) \end{array}$$ (4)

The one-class SVM is a supervised learning algorithm and requires a labeled training set for target class examples. To use one-class SVM with unsupervised learning, we employ an enhancement proposed in [38]. The enhancement considers the normal flows in the training dataset as outliers and removes them before training. The enhanced SVM introduces a variable *η* which represents an estimate that an instance in the unlabeled training set belongs to the target class (malicious flows) or is an outlier (normal flows). The *η* has value near to 0 for all outliers and eliminates the effect of outliers in the SVM training. Another variable *β* controls the maximum number of points that are allowed to be outliers. Using enhancement proposed in [38], the Eq (2) can be written as:

$$\begin{array}{c} min_{w,\rho }min_{\eta_{i}}\frac{1}{2}{\left\|w\right\|}^{2}+\\ \frac{1}{m\nu }\sum_{i=1}^{m}\eta_{i}max_{i}\left(0,\rho -w\phi \left(x_{i}\right)\right)-\rho \\ \text{subject}\;\text{to}\;e^{T}\eta \geq m\beta \end{array}$$ (5)

The minimization problem shown in Eq 5 is a non-convex problem which means that is very difficult to find a global minimum point. The problem is solved using the concave convex procedure [38]:

Let *g*(*h*(*w*)), where *h*(*w*) = *max*(0, *ρ* − *wϕ*(*x*)) and *g*(*u*) = *inf*_*β*∈0,1_[*β*^*T*^ *μ*], using concave duality, the objective function is reformulated as follows:

$$\begin{array}{c} \begin{array}{cc} min_{w,\rho ,\eta }E_{vex}+E_{cave}\\ E_{vex} & =\frac{\left||w|\right|}{2}+\eta h\left(w\right),E_{cave}=g^{*}\eta \end{array} \end{array}$$ (6)

where g* is the concave dual of *g*. *E*_*vex*_ and *E*_*cave*_ are concave and convex differentiable functions.

**Fig 4:**
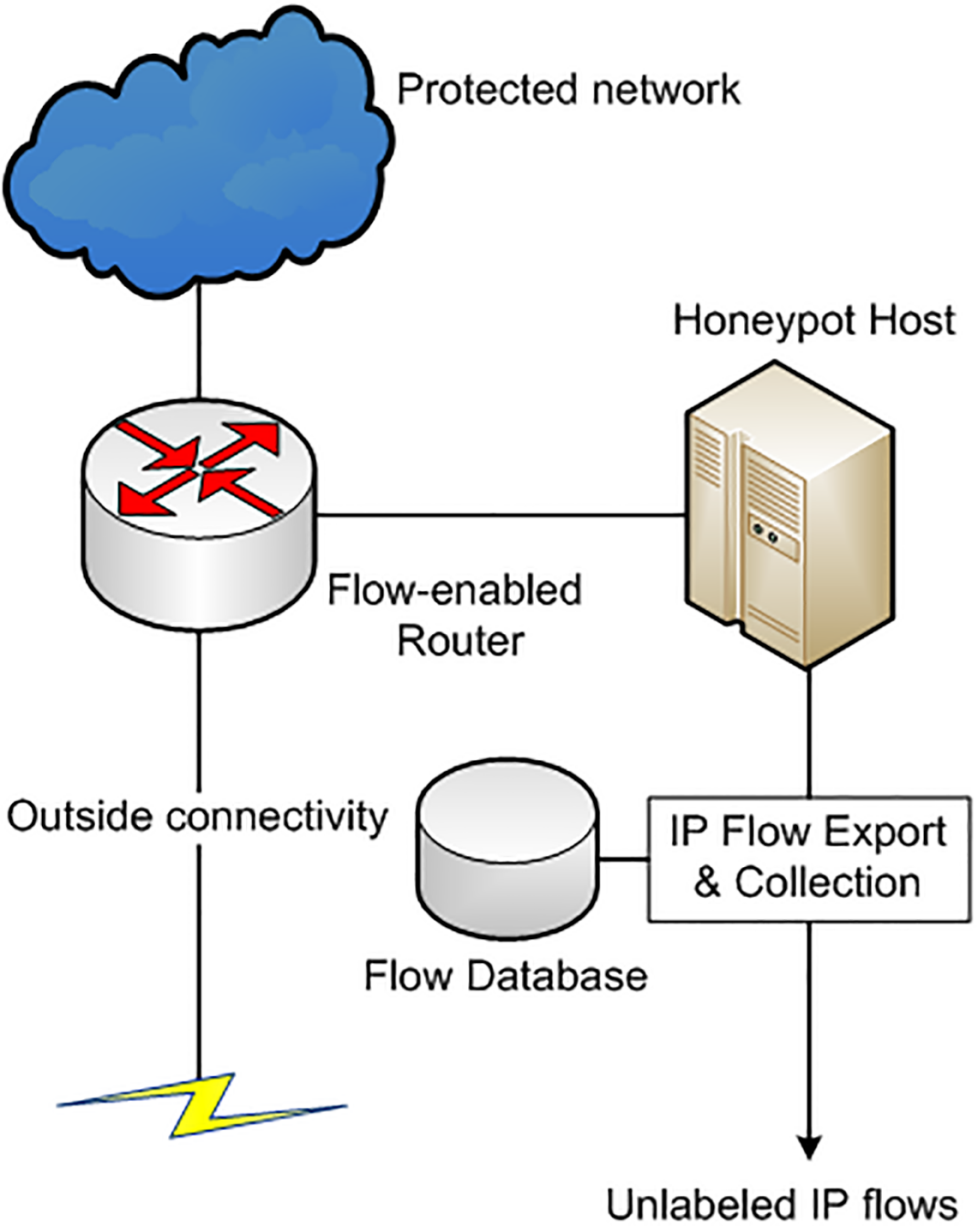
Malicious flow collection process.

**Fig 5:**
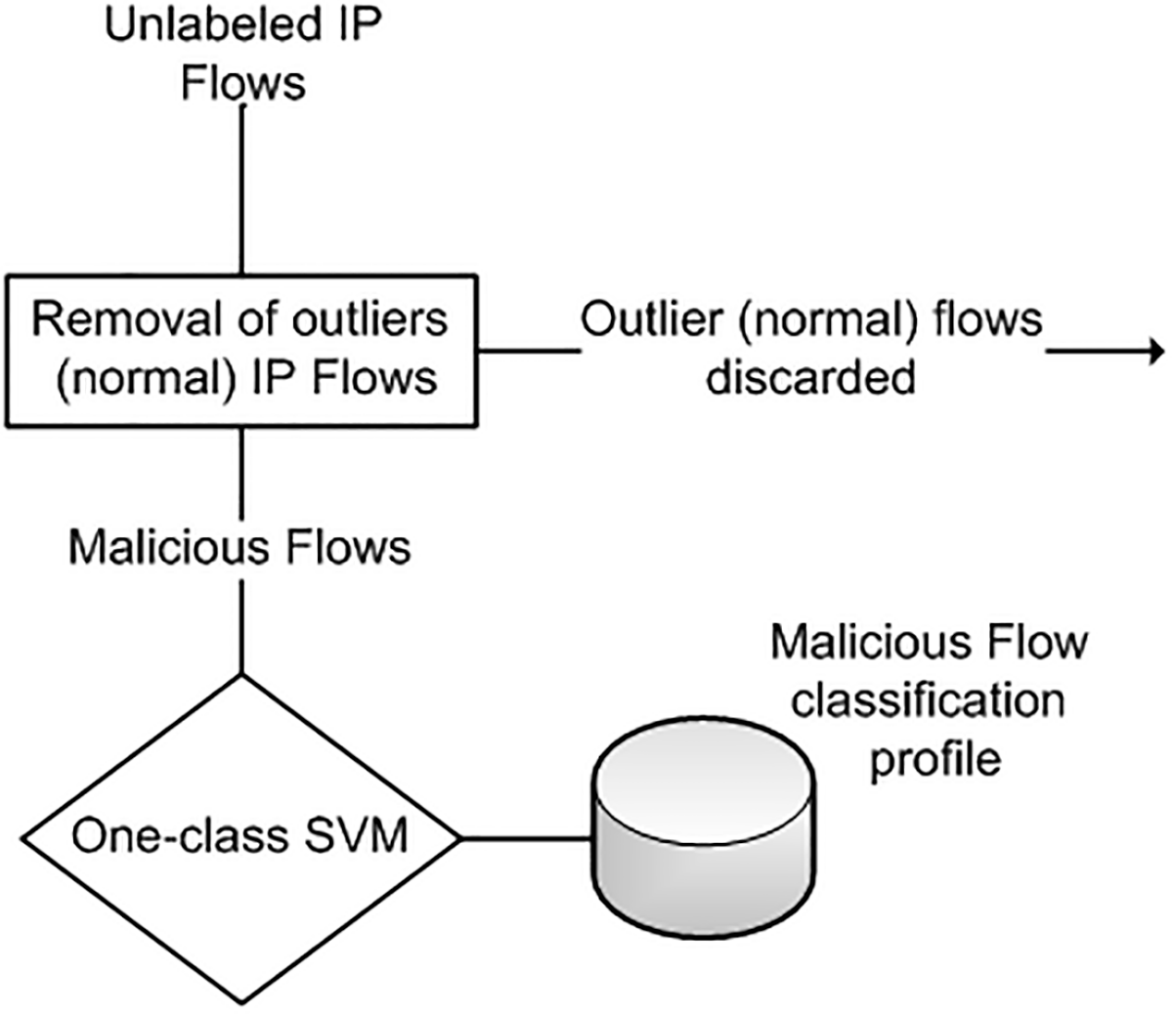
Training of one-class SVM using malicious flow.

The enhanced one-class SVM requires that malicious flows in unlabeled training dataset should be in sufficiently large quantity than normal flows. To ensure that majority of flows in the unlabeled training dataset are malicious, we propose the use of honeypot-based flow collection architecture to generate the unlabeled dataset for the training of one-class SVM [39]. Fig 4 shows the malicious flow collection process using honeypot. The honeypot is directly connected with the external routing interface. The flow records collected through honeypot are mostly malicious [40] and may also contain some non-malicious traffic. The unlabeled flows are forwarded to the one-class SVM classifier. These malicious flows are used for training of of enhanced one-class SVM as shown in Fig 5. The one-class SVM employs an outlier detection step which removes any non-malicious flows from the dataset. Only malicious flows are utilized to build a malicious flow classification profile.

After training, the one-class SVM is used to process the flows being extracted from the network. The one-class SVM separates malicious flows from the network traffic. The malicious flows are forwarded to the second stage detection process while normal flows are discarded.

### The second stage detection

The first stage detection process only separates malicious flows from the network. It does not associate an attack class with malicious flows. These malicious flows require a manual inspection to determine the attack type and corrective actions. Although malicious flows are in a small fraction as compared to normal network traffic, these flows can still be in large numbers in the NGN environment. Manual inspection of such large number of flow is a difficult task. To group similar malicious flows together, we employ the second stage detection process. The second stage detection process automatically place malicious flows into different attack clusters.

We use a self-organizing map (SOM) for clustering of malicious flows into different attack clusters [41]. The SOM is a neural network consisting of an input and output neuron layers. The neurons in the input layer inter-connects with neurons in output layer through unsupervised competitive learning network [42]. The competitive learning is a winner-take-all approach and consists of two steps; competition and cooperation. In the competition phase, a neuron in output layer is selected among all competing neurons using minimum Euclidean distance. The neuron whose weight vector comes the closest to the input vector is declared winner.

Mathematically, for each input *v* ∈ *V*, i* neuron is declared “winner” if:

$$\begin{array}{c} i^{*}=argmin_{i}\left|\right|w_{i}-v\left|\right| \end{array}$$ (7)

In cooperation, the weights of the winner and its neighboring neurons are adjusted using a time decay function. The effect of weight adjustment is high at the origin and decreases with the distance and time. The range of the neighborhood is defined by a Gaussian function:

$$\begin{array}{c} \sigma \left(t\right)=\sigma_{0}e^{\left(-2\sigma_{0}\frac{t}{t_{m}}\right)} \end{array}$$ (8)

where

*σ*_0_ = Initial value of neighborhood range

*t* and *t*_*m*_ = The current and maximum iteration respectively

*σ*(*t*) = The range of neighborhood at *t* stage.

After a winning neuron is selected, the weights of neighboring neuron vectors are adjusted:

$$\begin{array}{c} w_{i}\left(t+1\right)=w_{i}\left(t\right)+\eta \left(t\right)\sigma \left(t\right)\left(v-w_{i}\left(t\right)\right) \end{array}$$ (9)

In the above equation, *t* represents the current stage and *η*(*t*) is the learning rate. The continuous process of competition and cooperating marks the cluster on topographic self-organizing map. Each neuron on the output layer denotes the resultant clusters. The number of the output clusters has to be set before the clustering by a user defined parameter *k*.

The SOM requires a training set of malicious flows to create profiles for different attack clusters. We define the number of attack cluster and corresponding labels using the domain knowledge of the network environment. We manually give an attack label to all clusters in the SOM by analyzing the network flows in the cluster. During clustering process, all incoming flows are compared with all clusters and the label of the closest cluster is given to the malicious flow.

## The datasets

The proposed model has been evaluated on three flow-based datasets. The first dataset was developed in University of Twente and is publicly available [23]. We have created the second dataset ourselves by combining network flows of various malware and Advance Persistent Threats (APTs) with normal flow traffic. The third dataset consists of SIP traffic data. The flow records in all datasets are in Netflow v5 format. The Netflow v5 is a widely used flow export and collection protocol and supported by all major hardware manufacturers [43]. We have used 9-tuple flow records in the experiment. The details of attributes in the 9-tuple flow records is given in Table 1.

**Table 1:** Detailed attributes for Netflow v5 flow records.

Attribute	Description
Source IP	The source IP address
Destination IP	The destination IP address
Packets	Number of packets in flow
Octets	Number of bytes in flow
Duration	The duration of flow in milliseconds
Source Port	Source port number
Destination Port	Destination port number
TCP Flags	Cumulative OR of TCP flags
Protocol	The transport layer protocol such 6 = TCP, 17 = UDP

### Sperotto’s dataset

The Sperotto’s dataset consists of 14.2M flow records collected through a “Honeypot” deployment in University of Twente network [23]. The honeypot was directly connected to the internet to ensure maximum exposure to attacks. Three common services SSH, HTTP and FTP were run over the honeypot. Information about the flows is extracted from the log files of receptive services. Part of the traffic in the dataset is the side effect of alerts and is not considered malicious. During the flow collection, one hacker installed an IRC proxy over the honeypot. The traffic generated due to IRC is also non-malicious. The alert types and number of flows corresponding to each alert type are shown in Table 2.

**Table 2:** Detailed flows in Sperotto’s dataset.

Alert Type	No. of flows	Category
SSH	13942629	Malicious
FTP	13	Malicious
HTTP	9798	Malicious
AUTH-IDENT	191339	Side effect
IRC	7383	Side effect
OTHERS	18970	Side effect

The four time related attributes *start-time start-msec, end-time and end-msec* in the original dataset are computed to a single attribute of duration in milliseconds [22]. Also the dataset itself does not contain any normal traffic, we have included a large number of normal flows in the dataset. The normal flows have been collected by ourselves from a medium-size network of legitimate users. The behavior of users during the normal flow collection period include browsing web, streaming videos, online games and remote server access.

The Sperotto’s dataset is very large, therefore we have extracted a subset of network flows from the dataset using random sampling. Table 3 gives details of network flows in the training and test dataset. The training dataset contains 10000 malicious flows and 500 normal flows. The testing dataset consists of 11740 malicious and 124240 normal flows.

**Table 3:** Test and training dataset—Sperotto dataset.

Training dataset		Testing dataset	
Malicious	Normal	Malicious	Normal
10000	500	11740	124240

### APT and malware dataset

The Sperotto’s dataset has a limited variety of malicious traffic. Most of the malicious traffic only consists of SSH attacks flows. To evaluate the performance of the proposed IDS against modern attacks, we experimented with the latest malware and advance persistent threats (APTs). We have generated flow records for different malware and APTs using packet capture files obtained from Contagio Malware Dump (http://contagiodump.blogspot.com/). We have used Sality, Asprox, TBot and Nuclear malware traffic. The network flow records of these malware and APTs are combined with normal flow traffic used earlier with Sperotto’s dataset. Table 4 shows the details of flow records in training and test dataset. The training dataset contains 3524 malicious and 350 normal flow records while test dataset has 5286 and 24387 flow records.

**Table 4:** Test and training dataset—Malware and APT dataset.

Training dataset		Testing dataset	
Malicious	Normal	Malicious	Normal
3524	350	5286	24367

### SIP dataset

The third dataset is a labeled VoIP dataset consisting of SIP packet traces [44]. The dataset has two sets of SIP traces collected from two different VoIP testbed networks. The first testbed uses Asterisk PBX server and the second testbed uses the OpenSIP proxy with RADIUS servers. We have only considered the OpenSIPs traces for evaluation in our experiment. The testbed configuration includes OpenSIP proxy and-and normal and malicious traffic generators. The normal traffic is emulated by groups of VoIP bots. Each group of bots connects with the internal and external interface of the SIP proxy respectively. The malicious traffic is generated using the Inviteflood and Splitter attack tools. The dataset is available in the form of SIP packet traces. We have used ntops’ nProbes tool to extract Netflow v5 based flow records from the SIP packet traces. The details of flow records in the dataset are given in Table 5. Table 6 gives details of network flows in training and test dataset.

**Table 5:** Detail of flow records—SIP dataset.

Traffic Type	No. of flows	Category
InviteFlood SIP traffic	6496	Malicious
Splitter SIP traffic	3927	Malicious
Normal SIP traffic	7901	Normal

**Table 6:** Test and training dataset—SIP dataset.

Training dataset		Testing dataset	
Malicious	Normal	Malicious	Normal
2083	300	10423	7901

## Results and discussion

### Experimental setup

We have designed our experiment using R and the Waikato Environment for Knowledge Analysis (WEKA). In first step, we have applied the R tool to remove the outlier from one-class SVM training set. The majority of flows in the unlabeled dataset are malicious with some normal flows. The enhanced one-class SVM marks the normal flows in the training dataset as outliers and does not use them during learning. The self-organizing map(SOM) in the second stage detection process also uses malicious flows to create different attack clusters. We manually set the number of attack cluster in SOM to six using domain knowledge of the evaluation environment. Each alert cluster relates to specific attack types or a service that is under attack.

### Sperotto’s dataset results

In the first experiment, we have used Sperotto’s dataset for the evaluation. Both detection stages of the IDS use the dataset shown in Table 3 for learning. Table 7 shows the confusion matrix for outlier detection process in one-class SVM training. The one-class SVM successfully removes 98.40% normal flows from the training dataset. The remaining 9161 flows out of 10000 are used by the one-class SVM for learning the malicious behavior. The six attack clusters include the incoming and outgoing flow traffic for the SSH and HTTP services and two additional clusters for placement of unknown alerts and miss-classified network flows.

**Table 7:** Confusion matrix for outlier detection during one-class SVM training—Sperotto’s dataset.

Classified as	Malicious	Normal (Outliers)
Malicious	9161	839
Normal (Outliers)	8	492

We process the test dataset shown in Table 3 after learning the malicious flow behavior. In the first stage of the detection process, the one-class SVM marks 11730 flows as malicious out of 11740 total flows. The flows marked malicious also contain 1301 normal flows as false positives. The flows identified as malicious by one-class SVM in first stage are forwarded to second stage. The second stage detection process categorizes the flows in different attack clusters. The total number of flows marked malicious by first stage are 13031 including 1301 false positives. The first stage detection process has a detection rate of 89%. The SOM process all malicious flows and places them in the closest attack cluster. The clustering results and the actual number of flows in every cluster are given in Table 8.

**Table 8:** Clustering malicious flows in second stage process—Sperotto’s dataset.

Alert Cluster	Actual No of Flows	Flows in attack cluster
HTTP IN	2127	2154
HTTP OUT	2113	2085
SSH IN	4140	3992
SSH OUT	3360	4006
Other-I	0	770
Other-II	0	24
Total	11740	13031

**Fig 6:**
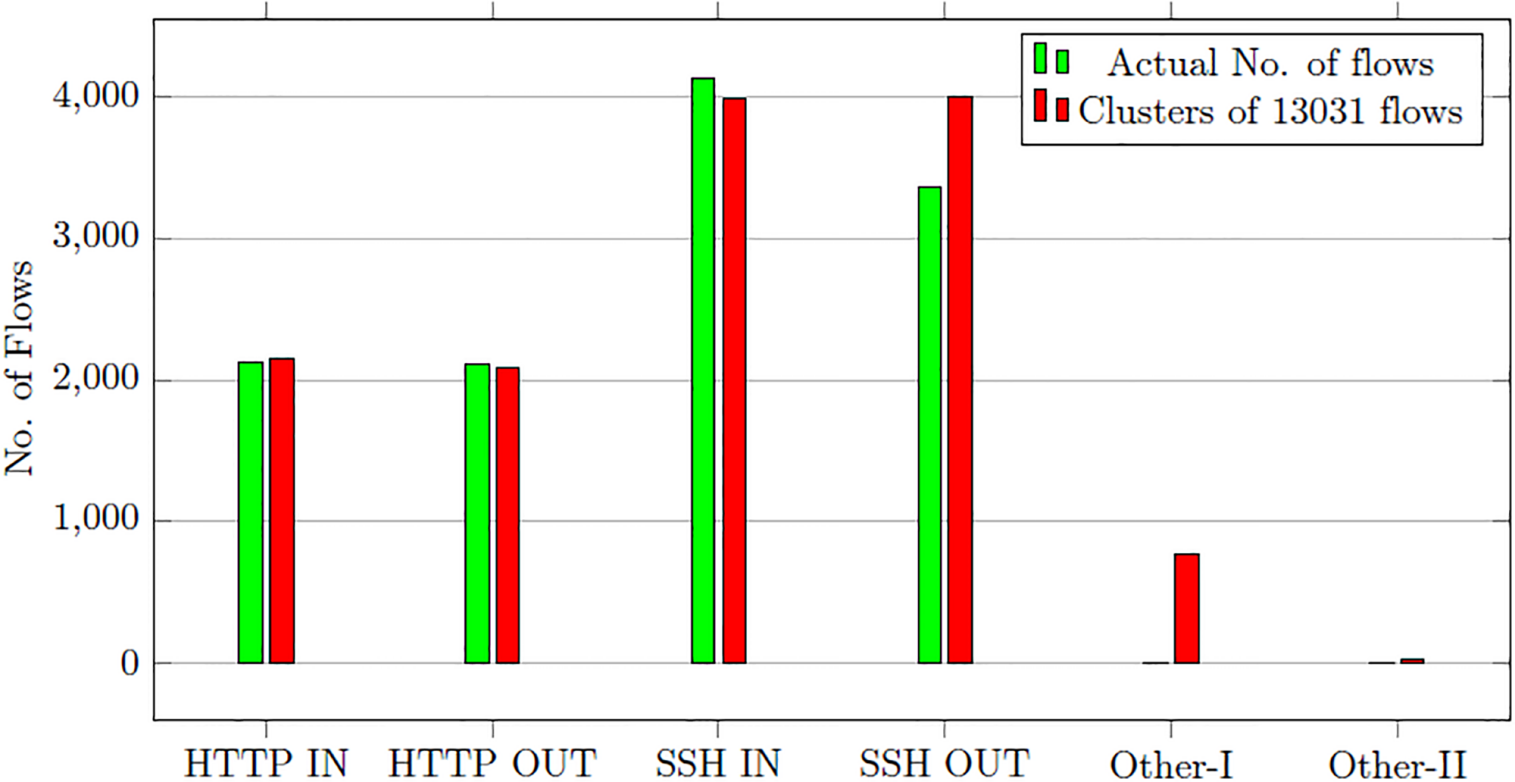
SOM clustering results comparison—Sperotto’s dataset.

The HTTP IN, HTTP OUT and SSH IN categories remain consistent and similar number of flows are available in the output clusters. The actual number of flows in HTTP IN category is 2127 while the output cluster contains 2154 flows. Therefore only 27 flows are placed incorrectly. The HTTP OUT cluster has 2113 flows in input dataset and its output cluster contains 2085 with 28 flows placed in the incorrect cluster. The SSH IN cluster has 4140 flows in the input set while output cluster contains 3992 flows. In this case, 148 flows have been incorrectly classified. The actual number flows for SSH OUT category is 3360 while the output cluster has 4006 flows. The SSH OUT category has the highest number of incorrectly classified flows i.e. 646. Also 770 and 24 flows are placed in Other-I and II clusters respectively. This relatively high rate of miss classification is due to the 1301 false positives (normal flows) of the first stage detection process. The comparison of clusters with actual flows in the input set is given in Fig 6.

### Malware and APT dataset results

In the second experiment, we have used malware and APT flow-based dataset for evaluation. The two-stage intrusion detection model is trained using the dataset shown in Table 4. The enhanced one-class SVM removes the normal flows from the training dataset leaving only the malicious flows. Table 9 shows the confusion matrix for outlier detection process in one-class SVM training. The one-class SVM successfully removes 94.28% normal flows from the training dataset. The remaining 2857 flows are used by the one-class SVM and SOM for learning the malicious behavior and creation of attack clusters.

**Table 9:** Confusion matrix for outlier detection during one-class SVM training—Malware and APT dataset.

Classified as	Malicious	Normal (Outliers)
Malicious	2857	330
Normal (Outliers)	20	667

The trained one-class SVM is presented with a test dataset of 29654 flows. It classifies 5226 flows as malicious out of total 5286 flows. The detected malicious flows also include 434 normal flows. The malicious flows detected in first stage are forwarded to SOM clustering algorithm in second stage detection process. The number of malicious flows is 5226 including 434 false positives. We manually set the number of attack cluster in SOM to six which include four clusters for malware and APTs and two additional clusters to place the un-clustered flows. The SOM places the malicious flows into closet matching attack clusters. The clustering results and the actual number of flows in every cluster are given in Table 10.

**Table 10:** SOM clustering results—Malware and APT dataset.

Alert Cluster	Actual Flows	Flows in attack cluster
Sality outgoing	1669	1312
Asprox outgoing	3336	3649
TBot outgoing	133	200
Nuclear outgoing	88	64
Other-I	0	2
Other-II	0	59
Total	5286	5226

**Fig 7:**
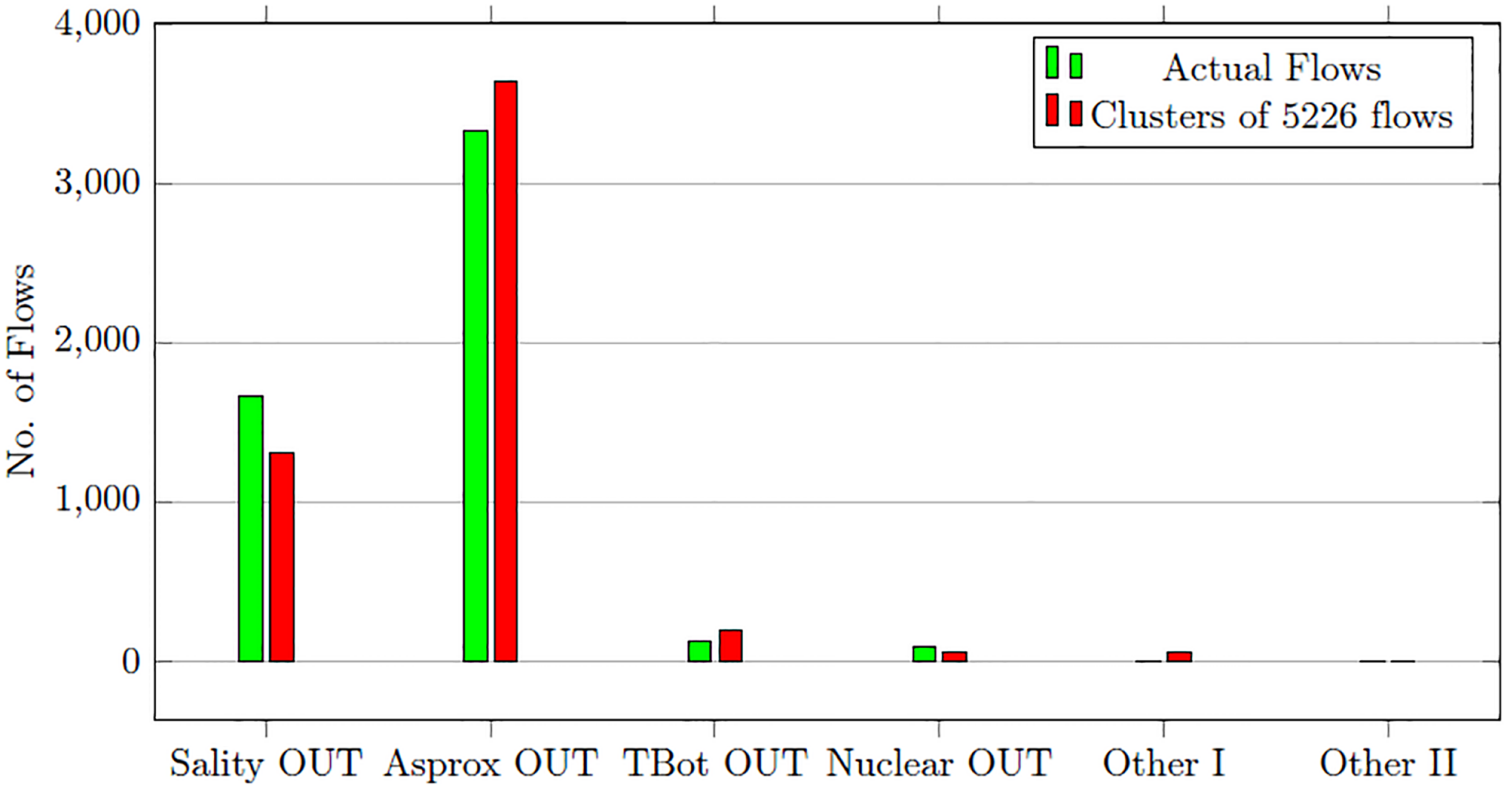
Malware and APT clustering results comparison.

1312 out of 1669 flows of Sality malware are placed in correct cluster. The Asprox attack cluster has 3649 flows while the actual number of flows is 3336. Some network flows of Sality malware are placed into Asprox cluster because Asprox malware traffic is not uniform. The false positives of the first stage detection process are separated into Other-I and Other-II clusters. Fig 7 compares the result of clustering with actual flows.

### SIP dataset results

In the third experiment, the SIP dataset given in Table 6, has been used for the evaluation. In the first stage detection, the enhanced one-class SVM uses the unlabeled training dataset for learning. The one-class SVM marks the normal flows as outliers and does not use them from learning. Table 11 shows the confusion matrix for outlier detection process. The one-class SVM successfully removes 94.05% normal flows from the training dataset. The remaining flows are used by the one-class SVM for learning the malicious behavior.

**Table 11:** Confusion matrix for outlier detection during one-class SVM training—SIP dataset.

Classified as	Malicious	Normal (Outliers)
Malicious	1701	91
Normal (Outliers)	30	170

**Fig 8:**
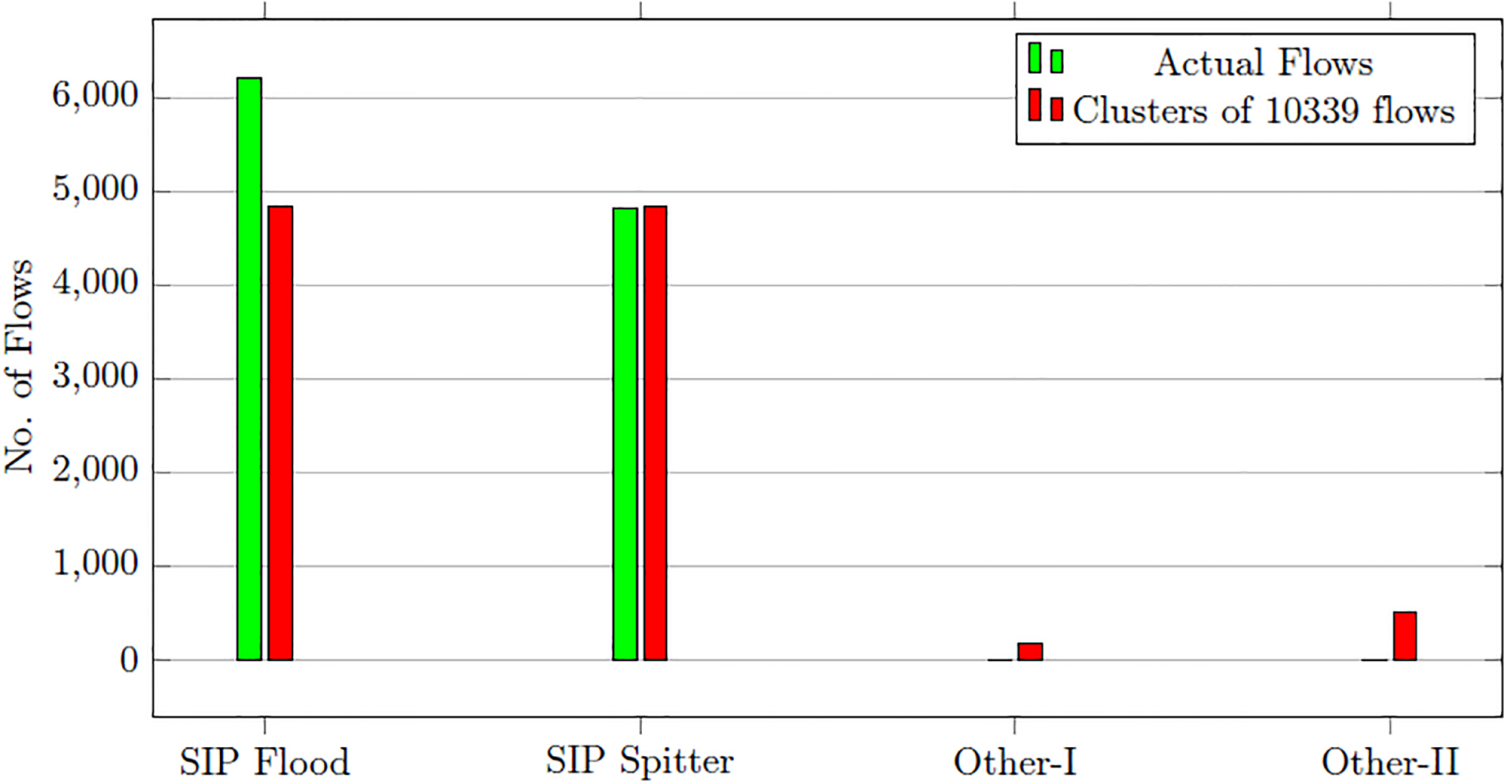
SOM clustering results comparison—SIP dataset.

After training, the one-class SVM process the test dataset. The one-class SVM correctly marks 10339 flows as malicious out of 10423 total malicious flows. There is no normal flow marked as malicious. The malicious flows identified in the first stage detection are forwarded to the second stage. The second stage uses the SOM for clustering of malicious flows according to attack types. We have used the same training dataset used in the first stage for training of SOM. Since there are two types of malicious flows in the dataset, we have set the number of clusters to four. The two additional clusters are used to contain the flows which SOM fails to associate with any attack type. The results of clustering process are given in Table 12. The first cluster consists of malicious flows belonging to the SIP flood. The SOM is able to cluster 4848 flows out of total 6224 flows. The actual number of in second attack cluster is 4815. However the resulting cluster consists of 4834 flows which also includes some flows belonging to first attack cluster. The number of un-clustered flows is 657 which are placed in Other-I and Other-II cluster. Fig 8 compares the results of clustered flows with actual flows.

**Table 12:** SOM clustering results—SIP dataset.

Alert Cluster	Actual Flows	Clustering results
SIP Flood	6224	4848
SIP Spitter	4815	4834
Other-I	0	162
Other-II	0	495
Total	10339	10339

### Discussion

We uses network flow records for intrusion detection in next-generation networks. The network flows consist of a fraction of complete network traffic. The use of network flow reduces the amount of data processed by the intrusion detection system. Therefore, our flow-based IDS is efficient as compared to traditional packet-based intrusion detection systems. Another advantage of flow-based inspection is independence of detection process from underlying protocols and network architecture.

We have designed a two-stage intrusion detection framework. The first stage uses a computational fast detection process and only recognizes malicious flows. In the first stage, the normal flows are discarded while malicious flows are forwarded to the second stage. Since malicious flows are in small quantity as compared to normal flows, the second stage can use a computationally expensive technique for the detailed intrusion detection. The intrusion detection process gives deep insights into the malicious traffic and associates an attack type with the malicious flows. The application of computationally expensive intrusion detection techniques is difficult in traditional single-stage detection systems due to the processing of both normal and malicious flows. Therefore the two-stage detection is efficient as compared to a single-stage detection.

The techniques used in both detection stages are based on unsupervised learning. Therefore, no labeled datasets are required for training of detection algorithms.

Our two-stage detection uses an enhanced one-class SVM in the first stage. One-class SVM techniques give better results for intrusion detection in malicious flow records. However, the accuracy of one-class SVM is very sensitive to the value of *ν* parameter [36]. The *ν* is an upper bound on the fraction of outliers (normal flows) and lower bound on the number of support vectors. We have experimented with different values of *ν* to obtain best possible results. The optimization of *ν* parameter is also a promising research area, and different techniques have been proposed to find out the optimal value of *ν* [45]. A limitation of enhanced one-class SVM is the requirement that malicious flows in the training set are in sufficiently higher than normal flows. In second stage detection, we have used SOM for automatic clustering of malicious flows. The results show that SOM correctly places the majority of flows in the correct cluster. However, domain knowledge of the traffic is required to determine the number and label of attack clusters. Our system uses unsupervised learning techniques, and no labeled datasets are required for training.

We have evaluated the proposed IDS on the three flow-based datasets. The results demonstrate that our proposed technique is accurate in the separation of malicious flows and grouping of malicious flows in different attack clusters.

## Conclusion

In this paper, we have proposed a two-stage flow-based intrusion detection model for next-generation networks. Next-generation networks provide voice, video and data services on a converged IP-based network. Our flow-based intrusion detection system is particularly useful in the context of next-generation networks (NGN) where different networks are converged to an all IP platform. Our proposed model processes the flow data in a two-stage detection process. The first stage uses a one-class SVM for efficient detection of malicious flows. The one-class SVM discards all normal traffics and forward the malicious traffic to second stage detection process. Due to the two-stage intrusion detection process, only malicious flows are analyzed in detail. Another important feature of our system is the use of unsupervised learning. The unsupervised learning does not need a labeled training datasets which are difficult to obtain for next generation networks. We have validated the approach on three flow-based datasets and results show that the proposed model gives promising results. In future, the proposed intrusion detection model can be implemented using additional flow attributes. The IPFIX /Netflow v9 define around 280 flow attributes which provide in-depth information about the network traffic. These additional attributes can be used to build intrusion detection schemes for detection of novel and stealth attacks.
